# Supplementary material for: Bscl2 Deficiency Does Not Directly Impair the Innate Immune Response in a Murine Model of Generalized Lipodystrophy
Source: J Clin Med. 2021 Jan 23;10(3):441. doi: 10.3390/jcm10030441 (PMC7865406; doi:10.3390/jcm10030441)
Supplement: Supplementary file 1 [file jcm-10-00441-s001.pdf]

**Supplementary Materials:**

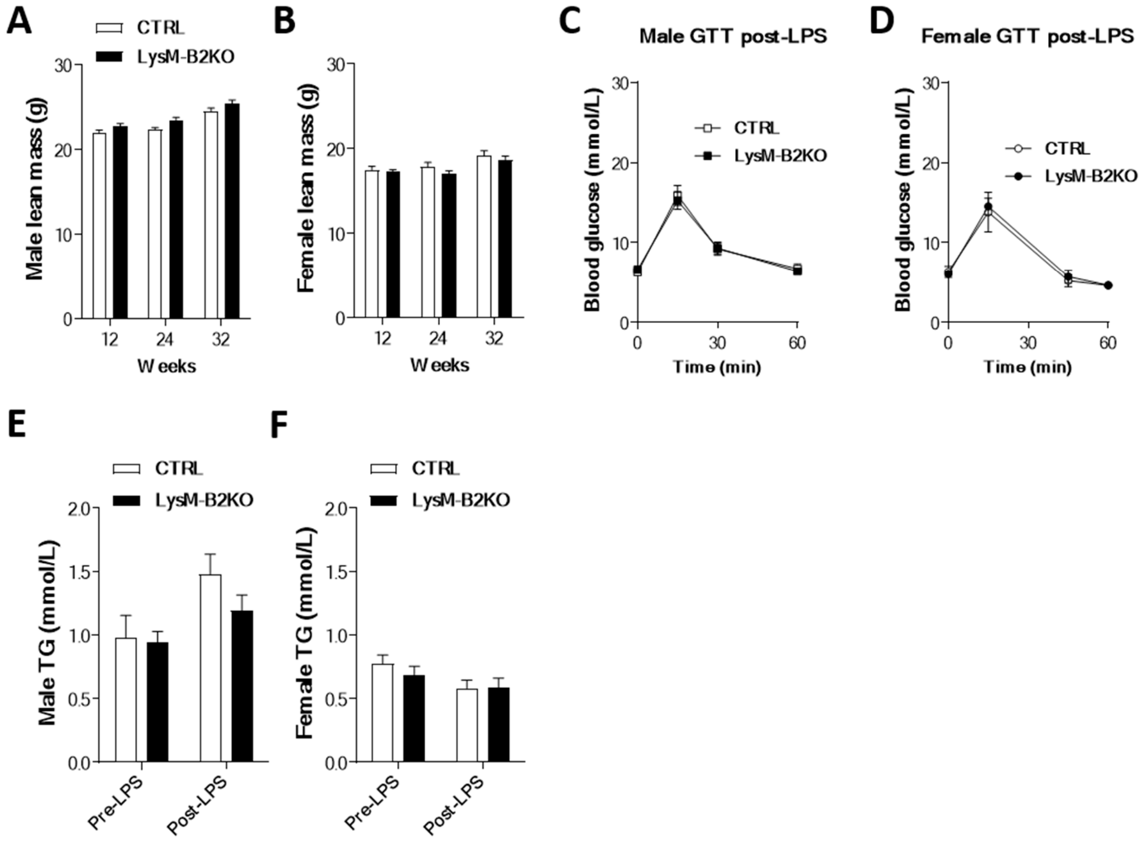


**Figure S1.** (**A–B**) Lean mass of male and female LysM-B2KO mice normalized to body weight (female n=5–7, male n=7–10). (**C–D**) Glucose tolerance of LysM-B2KO mice at 32 weeks of age 3 hours following intraperitoneally injection of 1mg/kg LPS. (**E–F**) Analysis of serum triglycerides in LysM-B2KO mice fasted for 5 hours and subjected to LPS treatment. Data are represented as mean ± SEM, $p$<0.05.

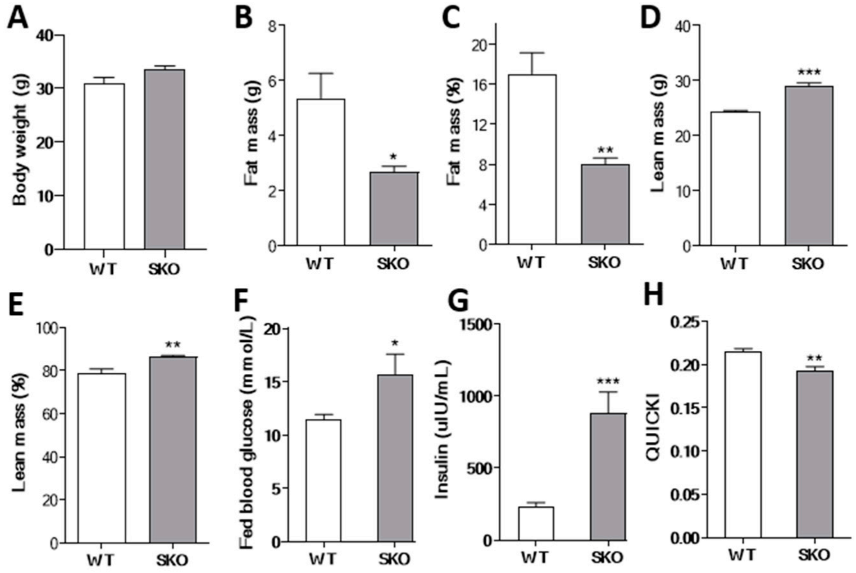


**Figure S2.** (**A–E**) Body composition of SKO mice. Body weight (**A**), fat mass (**B–C**) and lean mass (**D–E**) of male SKO mice at 16 weeks of age ($n$=5). (**F–H**) Serum analysis of SKO mice. Random fed blood

glucose levels (**F**), insulin (**G**) and quantitative insulin sensitivity check index (QUICKI) (**H**) analysis in SKO mice fasted for 5 hours. Data are represented as mean ± SEM, *$p$<0.05, **$p$<0.01, ***$p$<0.001.

**Table S1.** Table showing sequences or supplier details of qPCR primers used in this study.

| **Primer** | **Sequence** |
|---|---|
| NoNo F | GCCAGAATGAAGGCTTGACTAT |
| NoNo R | TATCAGGGGGAAGATTGCCCA |
| YWhaz F | GAAAAGTTCTTGATCCCCAATGC |
| YWhaz R | TGTGACTGGTCCACAATTCCTT |
| HPRT F | GTTAAGCAGTACAGCCCCAAA |
| HPRT R | AGGGCATATCCAACAACAAACTT |
| IL10 F | GCTCTTACTGACTGGCATGAG |
| IL10 R | CGCAGCTCTAGGAGCATGTG |
| TNF$\alpha$ F | CCCTCACACTCAGATCATCTTCT |
| TNF$\alpha$ R | GCTACGACGTGGGCTACAG |
| IL6 F | TAGTCCTTCCTACCCCAATTTCC |
| IL6 R | TTGGTCCTTAGCCACTCCTTC |
| IL1$\alpha$ F | GCACCTTACACCTACCAGAGT |
| IL1$\alpha$ R | AAACTTCTGCCTGACGAGCTT |
| IL1β F | GCAACTGTTCCTGAACTCAACT |
| IL1β R | ATCTTTTGGGGTCCGTCAACT |
| iNOS F | GGAGTGACGGCAAACATGACT |
| iNOS R | TAGCCAGCGTACCGGATGA |
| MCP1 F | TTAAAAACCTGGATCGGAACCAA |
| MCP1 R | GCATTAGCTTCAGATTTACGGGT |
| Bscl2 (TaqMan™ probes) | ThermoFisher assay ID #Mm01230976_m1 |
| 18s (TaqMan™ probes) | ThermoFisher assay ID #Mm03928990_g1 |
